# Utility of Multicellular Spheroids for Investigating Mechanisms of Chemoresistance in Triple-Negative Breast Cancer

**DOI:** 10.3390/ijms26157503

**Published:** 2025-08-03

**Authors:** Keith N. Ncube, Iman van den Bout, Clarissa Willers, Chrisna Gouws, Werner Cordier

**Affiliations:** 1 Department of Pharmacology, Faculty of Health Sciences, University of Pretoria, Pretoria 0007, South Africa; keith.ncube@up.ac.za; 2 Department of Physiology, Faculty of Health Sciences, University of Pretoria, Pretoria 0007, South Africa; iman.vandenbout@up.ac.za; 3 Centre of Excellence for Pharmaceutical Sciences (Pharmacen™), Faculty of Health Sciences, North-West University, Potchefstroom 2531, South Africa; clarissawillers@gmail.com (C.W.); chrisna.gouws@nwu.ac.za (C.G.); 4 NWU Desmond Tutu School of Medicine, Faculty of Health Sciences, North-West University, Potchefstroom 2531, South Africa

**Keywords:** chemoresistance mechanisms, multicellular spheroids, three-dimensional culture model, triple-negative breast cancer, two-dimensional culture model

## Abstract

Chemoresistance is a major challenge in the treatment of triple-negative breast cancer (TNBC). Multicellular spheroids are an attractive platform for investigating chemoresistance in TNBC, as they replicate the cues of the tumour microenvironment in vivo. We conducted a comprehensive literature search to summarise the multifactorial and interlinked mechanisms driving chemoresistance in TNBC spheroids. These mechanisms include spatial heterogeneity, hypoxia, extracellular matrix remodelling, tumour–stroma crosstalk, drug efflux, apoptotic resistance, and cancer stem cell signalling. Strategies for overcoming chemoresistance in TNBC spheroids include nanocarrier systems to overcome spatial diffusion limitations, pathway inhibition, and targeting tumour–microenvironment interactions. Despite their advantages, some spheroid models face challenges such as low reproducibility, a lack of heterogeneity, variability in size and shape, limited vascularisation, and constraints in long-term culture. Advanced culturing platforms such as clinostat bioreactors allow for extended culture periods, enabling mature spheroid drug testing. Furthermore, advanced analytical techniques provide spatially resolved spheroid data. These multifactorial and interlinked mechanisms reflect the tumour microenvironment in vivo that spheroids recapitulate, rendering them valuable models for studying chemoresistance. The incorporation of stromal components and advanced analytical workflows will enhance the utility and translational relevance of spheroids as reliable preclinical models for drug discovery in TNBC.

## 1. Introduction

In biological females, breast cancer is the most commonly diagnosed cancer and the leading cause of cancer-associated deaths [1]. There are various subtypes of breast cancer, and approximately 10–15% of cases are classified as triple-negative breast cancer (TNBC) [2]. The TNBC subtype is immunohistochemically defined as breast cancer that does not express human epidermal growth factor receptor 2/erb-b2 receptor tyrosine kinase 2 receptor and is both oestrogen-receptor- and progesterone-receptor-negative [3]. Characteristic traits of TNBC include an aggressive phenotype, poor prognosis, and high relapse rates compared to those for non-TNBCs [2,4]. Hormone- and growth-factor-receptor-targeted therapies are ineffective in TNBC, as this subtype lacks the overexpression of the target hormone receptors [5]. Surgery and chemotherapy, individually or in combination, remain the only management strategies available for TNBC patients [6]. The chemotherapeutic approaches used for the treatment of other main subtypes of breast cancer are also used in TNBC. Adjuvant chemotherapy is recommended for stage I (a tumour size > 0.5 cm) breast cancer [6]. Neoadjuvant chemotherapy is used in the early stages of the neoplasm with the goal of breast-conserving lumpectomy or for patients who temporarily cannot undergo surgery [7].

Despite a good (approximately 30% to 50%) pathological complete response (pCR) rate following treatment [8], TNBC patients who do not achieve a pCR are more likely to experience early recurrence and die from metastasis compared to non-TNBC patients [9]. Thus, a subset of TNBC patients benefits from chemotherapy, while approximately 50% become chemoresistant, leading to poor survival [10]. Multifaceted molecular mediators govern chemotherapeutic drug resistance in TNBC, and these include efflux transporters, cancer stem cells (CSCs) and associated signalling pathways, hypoxia, the avoidance of apoptosis, and microRNAs [11]. Although there have been substantial advancements in the characterisation of genetic alterations and molecular drivers of chemotherapeutic failure, oncology drugs are still associated with higher attrition rates compared to those for drugs for other ailments [12]. Various factors contribute to these high attrition rates, and the historical inability to efficiently translate preclinical research into clinical success is one of the main driving factors [13].

Many researchers have relied on traditional two-dimensional (2D) cell culture models for high-throughput preclinical drug screening [14]. In these models, the cells are cultured as monolayers on flat, solid culture surfaces such as plastic or glass [15]. The cells are forced to undergo cytoskeletal rearrangements, attaining artificial polarity, which results in aberrant gene and protein expression [16]. The cell-exchange-area-to-culture-media ratio in 2D models is increased, and the cells receive an excess supply of oxygen (O_2_) and nutrients. As a consequence, the O_2_, hormone, and nutrient gradients that are established in in vivo tumours are not reproduced, and cells are exposed to unrealistically high concentrations of drugs when conducting drug screening [17]. Multiple passaging of monolayer cells activates the process of immortalisation, and this leads to the selection of rapidly proliferating cells [18]. These cells do not adequately represent a tumour, as they are specifically sensitive to antiproliferative drugs [19]. Additionally, the tumour microenvironment (TME) is heterogenous with respect to the proliferative capacity of cells in different regions [20].

Due to the aberrant polarity, the orientation and clustering of surface receptors are altered, leading to the modification of the amount, composition, and configuration of the extracellular matrix (ECM) produced by monolayer cells [21]. Consequently, the cell-to-cell and cell-to-ECM interactions that occur in the TME in vivo are misrepresented [20]. These interactions play a significant role in tumour growth regulation, angiogenesis, aggression, and metastasis [22]. Additionally, continuous proteolytic digestion during the sub-culturing of monolayer cells results in cell surface protein alterations, which in turn lead to irregular cellular function [23]. Following trypsinisation, a culturing period exceeding two weeks is required for the re-establishment of physiological features mimicking those found in vivo [24], and this is challenging to conduct using 2D cell cultures. The 2D models do not optimally mimic the in vivo TME, and consequently, such findings can only partially be translated to in vivo models [25]. More representative platforms such as three-dimensional (3D) models offer an alternative to 2D models.

The presently available 3D platforms provide tissue-specific information at hierarchical levels of complexity, ranging from biologically complex animal models (including embryos and adults) and organ slices to simpler cell cultures grown in conditions that support 3D growth [26]. Examples of 3D models include tumour tissue explants, organ-on-a-chip, organoids, and multicellular spheroids. Spheroids are spherical microscale organotypic cell aggregates that often consist of stem cells, immortalised cell lines, or cells derived from human tumours (also referred to as tumouroids) [27]. Pioneered by Sutherland and colleagues [28] in the early 1970s, multicellular spheroids are a versatile model of advanced cell culturing in a 3D format [27]. As a result, multicellular spheroids have been used in basic research to probe the microenvironmental regulation of tumour-targeted drug delivery [29], energy metabolism [30], tumour dormancy [31], and cell–cell and cell–ECM interactions [32], amongst a wide array of other applications. There is an increasing quest to develop multicellular spheroids further and incorporate them into high-throughput drug screens, and this pursuit is driven by several desirable features of this culturing platform [27].

Spheroids mimic the complex architectural structure of the corresponding in vivo tissue better [33]. Specifically, spheroids maintain cell differentiation patterns analogous to those observed in tumours [33], and such patterns can be sustained over several weeks in culture [24]. These attributes are initiated and maintained by ECM deposition and intricate cell–cell and cell–ECM interactions found in vivo, which are not reproduced in conventional 2D culturing platforms [34]. Such biomimetic interactions foster the re-establishment of microenvironmental cues that could potentially impede drug delivery or that play a role in chemoresistance [29]. Relative to monolayers, multicellular spheroids also modify their gene and protein expression profiles to match the patterns observed in vivo [35,36]. Limitations in the mass transport of nutrients and metabolites in spheroids lead to spatial re-establishment of phenotypic zones similar to those found in the in vivo TME [37]. The spherical geometry of multicellular spheroids allows structure to be related to function, as the spatial gradients that develop within the spherical structures are correlated with alterations in cellular physiology [27]. Additionally, the spherical geometry of the spheroids allows for unsophisticated application of theoretical analyses, for example, using 2D projections of images to calculate the volume of spheroids as an experimental endpoint in drug studies [38]. Such an advantage is not readily offered by more complex 3D culturing platforms. Additionally, relative to other platforms, multicellular spheroids are cost-effective and easily maintained and can be genetically manipulated [39], rendering them appropriate models for high-throughput probing of chemoresistance.

Spheroids are known to be resistant to photo-, radio-, and chemotherapy [40]. Various studies have demonstrated attenuated drug sensitivity in breast cancer spheroid models relative to that in 2D-cultured cells [19,41]. Potential resistance mechanisms include (but are not limited to) the development of hypoxia, limited drug penetration, drug efflux, and spatial molecular and morphological heterogeneity [42]. Probing the mechanisms of resistance using multicellular spheroids can aid in elucidating the molecular drivers of chemoresistance and reinforce the utility of such models as a reliable platform for probing the in vitro efficacy of novel chemotherapeutic entities against TNBC. Therefore, we conducted a comprehensive literature review to explore the utility of spheroids in investigating the mechanisms of chemoresistance in TNBC. The Academic Search Complete (EBSCOHost), MEDLINE (via EBSCOHost), Web of Science Core Collection, and Scopus databases were searched. The search terms included combinations of “TNBC”, “chemoresistance”, and “spheroids”, along with the lineage names of commonly used TNBC cell lines. Relevant studies were selected based on their use of spheroid models to investigate chemoresistance in TNBC. Common mechanisms identified in the reviewed studies were grouped and are reported in this narrative review.

## 2. The Mechanisms of Chemoresistance in TNBC Spheroids

Numerous studies summarised in this review reveal that TNBC spheroids have been used to evaluate diverse mechanisms of resistance to various chemotherapeutic agents. Among the reviewed studies, MDA-MB-231 and its derivatives were the most frequently used TNBC cell lines for spheroid generation, followed by other human TNBC cell lines such as MDA-MB-468, BT-549, and BT-20 and the 4T1 murine cell line. Less commonly used models included SUM159, SUM1315, HCC70, HCC1806, and MDA-MB-157. The biological origin of these cell lines must be considered when conducting chemoresistance studies, as cells derived from metastatic sites (i.e., MDA-MB-231, MDA-MB-468, MDA-MB-157, SUM1315, and CRL2335) may exhibit a different drug response [43] compared to that in cells derived from primary sites (i.e., BT-20, BT-549, SUM159, HCC70, and HCC1806). The metastasis status of each cell line was verified using the Cancer Dependency Map portal [44]. The widespread use of the MDA-MB-231 cell line can be attributed to its well-characterised mesenchymal-like phenotype, high invasiveness, and suitability for both in vitro and in vivo studies [45,46]. Other cell lines like MDA-MB-468 and BT-20 offer distinct biological features that may be suited to specific research aims. When selecting a cell line in which to investigate the mechanisms of chemoresistance, careful consideration is essential, as spheroids generated from different lines vary in their morphology and compactness [47]. These structural differences can influence cellular responses to treatment and affect the interpretation of the mechanism of action. Additionally, phenotypic differences invariably alter the model’s suitability for specific chemoresistance pathways, for example, those mediated by the presence of specific drug targets or prone to unique cancer hallmarks.

**Figure 1:**
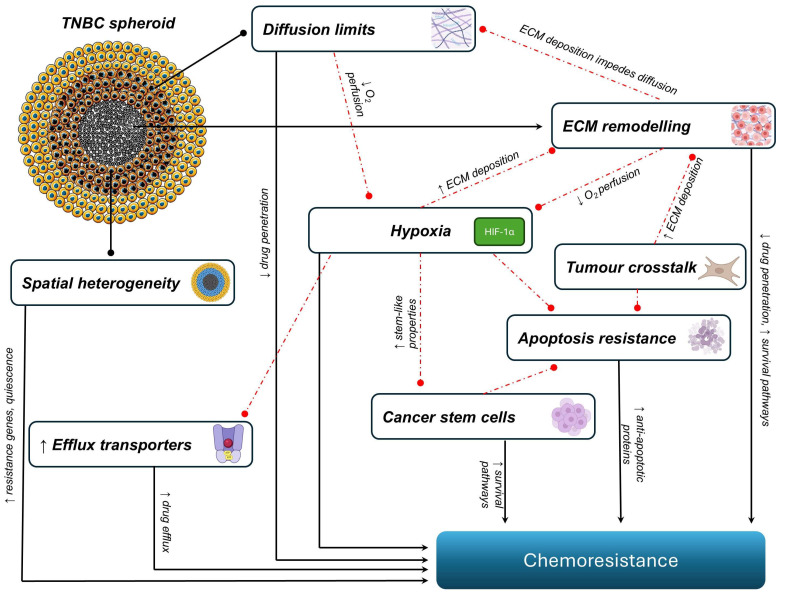
The most commonly investigated mechanisms contributing to chemoresistance in triple-negative breast cancer spheroids. The schematic shows interconnected factors that drive resistance to chemoresistance in spheroids. These include spatial heterogeneity, with cellular quiescence and regional variability in proliferation; extracellular matrix (ECM) remodelling, with increased ECM components such as collagen limiting drug penetration; apoptotic resistance, where the upregulation of anti-apoptotic factors impairs chemotherapy-induced cell death; tumour crosstalk, where the interaction with cancer-associated fibroblasts (CAFs) and tumour-associated macrophages (TAMs) promotes a pro-survival microenvironment; cancer stem cells (CSCs) and molecular pathways, with the upregulation of stemness markers and signalling pathways; hypoxia, where a limited O_2_ supply leads to the stabilisation of HIF-1α (hypoxia-inducible factor 1-alpha); and drug efflux, where increased expression of efflux pumps such as P-glycoprotein (P-gp) reduces intracellular drug accumulation.

The most frequently studied mechanisms of chemoresistance using TNBC spheroids include spatial heterogeneity; diffusion limits; hypoxia; ECM remodelling; tumour crosstalk; drug efflux; resistance to apoptosis; cancer stem cells; and pathway-driven resistance (Figure 1). These mechanisms should not be considered as isolated processes but rather a complex integration of the microenvironment, permitting multiple resistance pathways to emerge and interact. For instance, ECM remodelling can affect drug diffusion, contributing to hypoxia and favouring the survival of apoptosis-resistant or stem-like cells. This interconnectedness demonstrates how spheroids support a more accurate and multifaceted replication of tumour behaviour, particularly in the context of chemoresistance.

### 2.1. Spatial Heterogeneity

The limited diffusion of O_2_ and nutrients, especially in spheroids exceeding a diameter of 500 µm, leads to the development of spatially heterogeneous regions, comprising a proliferative rim, a quiescent zone, and a necrotic core [48]. In addition to spatial morphological [19] differences and cellular kinetic differences, distinct signalling pathways have been observed in different regions of TNBC spheroids. For example, the core of TNBC spheroids is enriched with drug-resistant stem-like cells expressing hypoxia-inducible factor (HIF)-driven stemness markers such as cluster of differentiation (CD)24, CD133, Nanog [49], and aldehyde dehydrogenase 1 family member (ALDH1A3)-positive CSCs that are more frequently found in the core than at the rim [50]. The quiescent cells in the spheroid core are inherently chemoresistant to cycle-dependent drugs, while the dense, ECM-rich outer layer impedes the penetration of chemotherapy [48]. Therefore, spatial heterogeneity in spheroids not only affects cellular phenotypes but also contributes to differential drug responses [51].

**Figure 2:**
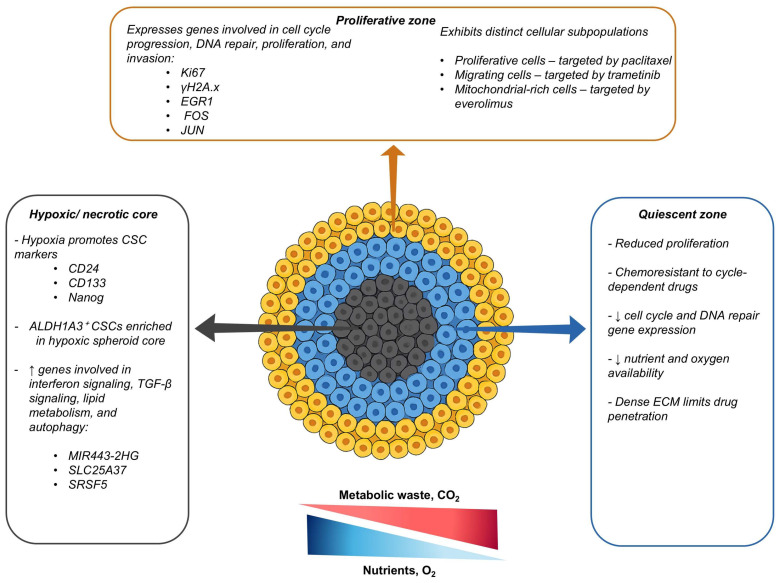
A schematic representing how spatially distinct zones contribute to chemoresistance in TNBC spheroids. The outer proliferative zone expresses genes involved in cell cycle progression, DNA repair, proliferation, and invasion (Ki67, γH2A.x, EGR1, FOS, JUN) and contains subpopulations differentially targeted by paclitaxel (proliferative cells), trametinib (migrating cells), and everolimus (mitochondrial-rich cells). The quiescent zone has reduced proliferation rates, downregulation of cell cycle and DNA repair genes, resistance to cycle-dependent drugs due to oxygen and nutrient availability, and a dense extracellular matrix (ECM) that limits drug penetration. The hypoxic/necrotic core promotes the expression of cancer stem cell (CSC) markers (CD24, CD133, Nanog), enrichment in ALDH1A3^+^ CSCs, and the upregulation of genes associated with interferon signalling, transforming growth factor-beta (TGF-β) signalling, lipid metabolism, and autophagy (MIR443-2HG, SLC25A37, SRSF5). Gradients of nutrients and metabolic waste (e.g., CO_2_) promote spatial heterogeneity and consequent chemoresistance within the spheroid.

Spatial transcriptomic profiling has recently revealed distinct cellular phenotypes in different regions of MDA-MB-231 spheroids [51]. Genes regulating stress adaptation, lipid metabolism, and autophagy are upregulated in the spheroid core [51]. In contrast, genes regulating the cell cycle, homology-directed repair of double-stranded DNA breaks, and the cellular senescence cycle are limited to the periphery, where cells exhibit higher proliferative indices relative to those at the core [51]. Relative to monolayers, these spheroids have increased resistance to paclitaxel and etoposide, likely due to the protective effects of the spheroid core and its associated stress adaptation mechanisms [51,52]. Using an optimised 3D whole-spheroid imaging approach in MDA-MB-231 TNBC spheroids, Mahmoud et al. [52] identified three spatially distinct subpopulations comprising proliferative cells, migrating cells, and cells with a high mitochondrial mass [52]. These phenotypes were localised to the outer spheroid regions and exhibited differential drug sensitivities, with paclitaxel selectively targeting dividing cells, trametinib targeting migrating cells, and everolimus targeting mitochondrial-rich cells [52]. However, no single agent effectively eliminated all phenotypes, allowing some subpopulations to survive treatment and eventually drive chemoresistance [52]. Collectively, these studies demonstrate that the spatial heterogeneity in TNBC spheroids leads to the emergence of region-specific cellular phenotypes with distinct survival advantages, resulting in differential drug sensitivities and sustained chemoresistance. Figure 2 illustrates how spatially distinct zones in TNBC spheroids contribute to differential drug responses and persistent resistance.

### 2.2. Diffusion Limits and Hypoxia

Compared to monolayers, the third dimension in multicellular spheroids confers structural barriers that can limit drug penetration, leading to chemoresistance. This has been demonstrated in MDA-MB-231 spheroids, where free doxorubicin was largely restricted to the spheroid periphery, whereas delivery via a nanocarrier system improved penetration and enhanced doxorubicin’s efficacy [53]. Imamura et al. [41] showed that TNBC cell lines that form dense spheroids are relatively resistant to doxorubicin and paclitaxel in comparison to those that form loosely packed spheroids [41]. Additionally, larger MDA-MB-157 spheroids are more resistant to doxorubicin treatment relative to smaller spheroids from the same cell line [49]. Collectively, these studies highlight that spheroid architecture plays a role in limiting the drug distribution and promoting chemoresistance. The increased density and size of spheroids not only hinder drug diffusion but also lead to the development of hypoxic cores, which contribute further to treatment resistance through various mechanisms [41]. Importantly, the formation and extent of hypoxic or necrotic cores are also influenced by the spheroid generation method. For example, in dynamic cultures, an improved distribution of oxygen and nutrients tends to reduce hypoxic core formation relative to that under static culturing conditions [54].

In addition to the spatial distribution of cellular phenotypes and structural barriers, there can be spatial differences in the oxygenation status of different spheroid regions [49]. Using pimodazole staining and carbonic anhydrase IX expression, it has been shown that larger spheroids in the MDA-MB-157 cell line develop a hypoxic core, while smaller ones do not [49]. In cancer, HIFs play a role in enabling the tumour cells to adapt to low-oxygen conditions by regulating genes involved in survival, metabolism, and therapeutic resistance [55]. In spheroids, the hypoxic core can induce HIFs, reduce the proliferation rate of the cells in the spheroid core, and upregulate stem cell markers, all of which collectively contribute to chemoresistance [49]. Reduced proliferation may limit the effectiveness of cell-cycle-targeting drugs, which require active cell division to exert their cytotoxic effects. For example, the expression of HIF-1α and resistance to doxorubicin are higher in MDA-MB-231 spheroids, showing an approximately 10-fold increase in the half-maximal inhibitory concentration (IC_50_) compared to that in their monolayer counterparts [56]. The increased expression of HIF-1α in spheroids is associated with an increase in stemness markers and immunosuppression factors such as indoleamine 2,3-dioxygenase 1 and transforming growth factor-beta (TGF-β), which remain elevated despite doxorubicin treatment [56]. This suggests that hypoxia in TNBC spheroids can contribute to chemoresistance and potentially promote immune-evasive cellular phenotypes [56]. Additionally, the induction of HIF-1α signalling using extracellular adenosine triphosphate (ATP) promotes chemoresistance to cisplatin in TNBC spheroids [57]. Corroborating these findings, hypoxia in MDA-MB-231 spheroids results in a 2-3-fold increase in the IC_50_ of cisplatin compared to that in spheroids in normoxic conditions [57]. Collectively, these findings demonstrate that hypoxia and associated HIF-1α signalling are cardinal physiological traits of spheroids that contribute to chemoresistance in TNBC spheroids. Relative to other breast cancer subtypes, TNBC is commonly associated with increased HIF-1α levels, and consequently, HIF-1α has emerged as a promising drug target in TNBC [58].

In solid tumours, HIFs have been shown to influence other physiological alterations that contribute to chemoresistance [59]. These include the upregulation of efflux transporters [60], reduced apoptotic sensitivity [61], the emergence of stem-like phenotypes [62], alterations in DNA repair [63], immune modulation, and the induction of cellular quiescence [59]. Rather than acting in isolation, these adaptations occur in parallel and are often reinforced by changes in the TME. The architecture and capacity of spheroids to develop hypoxia render these models a useful platform for investigating the interplay of these mechanisms under more representative conditions. Among these microenvironmental adaptations, hypoxia-induced remodelling of the ECM is an additional key contributor to chemoresistance [64].

### 2.3. Extracellular Matrix Remodelling

Similarly to in vivo tumours [65], abundant stromal proteins stiffen the ECM and impede drug diffusion in multicellular spheroids. For example, the hypersynthesis and crosslinking of collagen in TNBC spheroids restrict drug penetration, preventing both small molecules and nanoparticles from reaching the core [66,67]. Tezcan et al. [66] demonstrated that doxorubicin-resistant 4T1 TNBC spheroids developed an ECM-rich microenvironment that markedly reduced the penetration of polymeric and liposomal carriers [66]. Similarly, heterospheroids consisting of MDA-MB-231 cells, co-cultured with various stromal cells, have been shown to exhibit a high ECM content, which leads to reduced drug uptake and efficacy [67]. These studies highlight that ECM remodelling in spheroids physically hinders drug diffusion, contributing to chemotherapeutic resistance.

In addition to acting as a physical barrier, the ECM activates pro-survival signalling pathways via integrins and other ECM components. Integrins play a central role in mediating chemoresistance in TNBC by regulating survival signalling pathways and drug response mechanisms [68]. For instance, Lovitt et al. [69] demonstrated that TNBC spheroid cells grown in Matrigel^®^ exhibited an approximately 7-fold increase in their resistance to doxorubicin relative to that in their monolayer counterparts [69]. This chemoresistance is attributed to cell–ECM interactions mediated by β1 integrin signalling, which leads to the upregulation of the pro-survival proteins B-cell lymphoma 2 (Bcl-2) and B-cell lymphoma-extra-large (Bcl-xL) [69]. In this study, the inhibition of β1 integrin signalling restored drug sensitivity and reduced spheroid viability [69]. Cells on the spheroid periphery in direct contact with the ECM expressed high Bcl-2/Bcl-xL levels, suggesting that integrin engagement confers an anti-apoptotic advantage, leading to an attenuated drug response [69].

While integrin–ECM interactions drive resistance through survival signalling, the adhesion profile in TNBC spheroids also differs from that in 2D cultures, further contributing to chemoresistance [70]. In monolayers, the cells primarily engage in integrin-mediated cell–substrate adhesion, whereas cadherin-dependent cell–cell adhesion predominates in spheroids [70]. This shift alters the intracellular signalling and strengthens the spheroids’ structural integrity, thereby limiting drug penetration and efficacy [70]. For example, in BT-20 TNBC monolayers, the compound EE-15-one disrupts integrin-based focal adhesions and reduces cell viability [70]. However, BT-20 spheroids remain largely unaffected, with no significant changes in their volume or structural integrity following treatment [70]. The resistance observed in spheroids is likely due to the preservation of cadherin-mediated adhesion, which is not targeted by this agent [70]. This highlights how the transition from integrin- to cadherin-dominant adhesion in TNBC spheroids contributes to chemoresistance.

Additionally, the incorporation of the decellularised tumour ECM into MDA-MB-231 spheroids enhances their chemoresistance by activating the phosphoinositide 3-kinase (PI3K)/Akt/mTOR pathway, a signalling cascade known to support cell survival and resistance to cytotoxic agents [71]. Compared to standard spheroids, the ECM-enriched spheroids exhibited reduced sensitivity to cisplatin, gemcitabine, and palbociclib, further elucidating the role of the ECM in mediating chemoresistance in TNBC spheroids [71]. Together, these studies reveal that the ECM, an important element of the TME, not only hinders drug diffusion, contributing to chemoresistance in TNBC spheroids, but also activates pro-survival signals, promoting drug-resistant cellular phenotypes. These findings demonstrate that multiple ECM-derived signalling pathways, including β1 integrin and PI3K and Akt and mTOR, contribute to drug resistance in TNBC spheroids. Beyond ECM-mediated mechanisms, stromal cells within the TME can also promote chemoresistant phenotypes.

### 2.4. Tumour–Stroma Crosstalk and Microenvironmental Signalling

**Figure 3:**
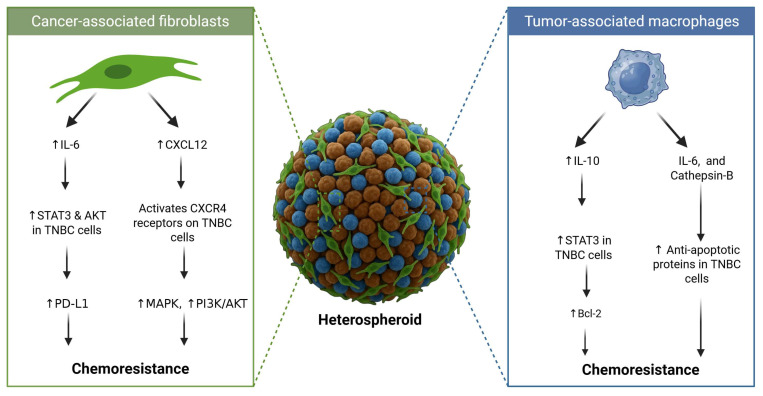
Cancer-associated fibroblasts (CAFs) contribute to chemoresistance in triple-negative breast cancer (TNBC) in two ways: through (i) the secretion of interleukin-6 (IL-6), leading to increased STAT3 and AKT activation and the subsequent downregulation of programmed cell death ligand 1 (PD-L1), and (ii) the secretion of C-X-C motif chemokine ligand 12 (CXCL12), which activates chemokine receptor type 4 (CXCR4) receptors on the TNBC cells, leading to the upregulation of the mitogen-activated protein kinase (MAPK) and phosphatidylinositol 3-kinase (PI3K)/protein kinase B (AKT) signalling pathways. Tumour-associated macrophages (TAMs) promote chemoresistance in TNBC cells via the (i) secretion of interleukin-10 (IL-10), which increases STAT3 activation, leading to elevated Bcl-2 levels, and (ii) the co-secretion of IL-6 and cathepsin-B, resulting in an increase in pro-apoptotic proteins, leading to chemoresistance.

The TME plays an important role in mediating chemoresistance in TNBC, with the stromal cells acting as more than just mechanically supportive structural elements [72]. In addition to the ECM, surrounding stromal cells such as cancer-associated fibroblasts (CAFs) and tumour-associated macrophages can also contribute to chemoresistance [73]. As such, co-culturing CAFs with TNBC cell lines propagated as spheroids allows for the recapitulation of the heterotypic nature of the in vivo TME. This facilitates tumour crosstalk that regulates the activation of survival pathways that lead to chemoresistance [73]. Specifically, the interleukin (IL)-6 secreted by patient-derived CAFs has been shown to activate the signal transducer and activator of transcription 3 (STAT3) and the protein kinase B (AKT) signalling pathways, leading to the upregulation of programmed cell death ligand 1 (PD-L1) and consequent resistance to doxorubicin [73]. This upregulation of PD-L1 additionally reduced the killing effect of folate receptor alpha-targeted chimeric antigen receptor T-cell therapy in spheroids from the MDA-MB-231 and HCC70 TNBC cell lines [73]. Moreover, co-culturing of chemokine receptor type 4 (CXCR4)-positive MDA-MB-231 TNBC cells with CAFs in spheroids results in resistance to paclitaxel [74]. Following treatment, the mitogen-activated protein kinase (MAPK) and PI3K pathways remain elevated in these spheroids, promoting proliferation and survival [74]. This proliferation and survival are mediated by the secretion of the C-X-C motif chemokine ligand 12 (CXCL12) by the CAFs, which subsequently binds to the CXCR4 receptors on TNBC cells [74]. Therefore, in addition to IL-6, CAFs can drive resistance in TNBC spheroids through CXCL12-CXCR4 signalling [74]. This demonstrates that the crosstalk between neoplastic cells and paracrine signals from CAFs contributes to both chemoresistance and immune evasion in TNBC spheroids (Figure 3) [73].

In addition to CAFs, tumour-associated macrophages (TAMs) also contribute to chemoresistance in TNBC spheroids (Figure 3). For instance, the incorporation of M2-like TAMs into MDA-MB-231 spheroids results in a greater than 2-fold increase in the resistance to doxorubicin compared to that in homotypic spheroid counterparts [75]. This chemoresistance is attributed to the macrophage-mediated release of cytokines such as IL-6 and enzymes such as cathepsin-B that activate anti-apoptotic pathways [75]. Similarly, heterospheroids consisting of MDA-MB-231 co-cultured with RAW 264.7 macrophages are more resistant to paclitaxel than cancer-cell-only spheroids [76]. In this case, the chemoresistance in the heterospheroids is associated with M2/TAM polarisation of the macrophages, evidenced by the increased secretion of IL-10 [76]. Additionally, epidermal growth factor (EGF) signalling between the cancer cells and the TAMs increased metabolic activity, potentially contributing to chemoresistance [76]. In addition to this, BT-20 spheroids embedded with TAMs express higher IL-10 levels compared to those in spheroids without TAMs [77]. This elevated IL-10 activates the IL-10/IL-10 receptor/STAT3/Bcl-2 signalling pathway, leading to chemoresistance to doxorubicin in these heterospheroids [77]. This IL-10/IL-10 receptor/STAT3/Bcl-2 signalling modulates doxorubicin resistance through the upregulation of anti-apoptotic proteins (Bcl-2) and ECM remodelling through collagen IV deposition [77].

This demonstrates that chemoresistance in TNBC spheroids is not solely mediated by intrinsic factors in cancer cells but is also influenced by paracrine signalling from the surrounding stromal cells. When properly optimised, the use of TNBC spheroids co-cultured with stromal cells can provide a valuable platform for elucidating the tumour-crosstalk-mediated mechanisms of chemoresistance. Beyond microenvironmental interactions, chemoresistance in TNBC spheroids can be driven by cellular mechanisms such as the overexpression of drug efflux transporters.

### 2.5. Drug Efflux

In addition to microenvironmental cues, the overexpression of multidrug-resistant drug efflux transporters, mainly P-glycoprotein (P-gp), also contributes to chemoresistance in TNBC spheroids. These ATP-binding cassette (ABC) transporters are commonly expressed by various cancers and actively pump drugs out of cells, thereby reducing intracellular drug accumulation and therapeutic efficacy [78]. Using a LightSpot ^®^-FL-1 fluorescent probe to visualise and quantify the P-gp expression in SUM1315 TNBC spheroids, a marked increase in the expression of P-gp was observed following treatment with olaparib, a poly (ADP-ribose) polymerase inhibitor [79]. There was a 33% increase in the P-gp expression in olaparib-exposed spheroids relative to that in untreated controls, highlighting the role of the P-gp transporters in mediating chemoresistance in TNBC spheroids [79]. More recently, single transcriptomic profiling of SUM159 TNBC spheroids resistant to doxorubicin and paclitaxel revealed the upregulation of genes associated with ABC transporter function [80]. Specifically, the ABCB1 gene that encodes for P-gp was increased 11-fold in the paclitaxel-resistant spheroids and 23-fold in the doxorubicin-resistant spheroids relative to its levels in drug-sensitive spheroids [80]. These studies affirm that P-gp is cardinal in mediating chemoresistance in TNBC spheroid models. Complementing drug efflux activity, other intrinsic survival mechanisms can potentiate chemoresistance in TNBC spheroids.

### 2.6. Apoptotic Resistance

In TNBC spheroids, the upregulation of anti-apoptotic mechanisms also contributes to reduced sensitivity to chemotherapeutic agents. The overexpression of anti-apoptotic proteins such as BCL2, Bcl-xL [81], and myeloid cell leukaemia 1 (MCL1) [82] contributes to reduced drug sensitivity in TNBC spheroids. Doxorubicin-resistant MDA-MB-231 spheroids rely on BCL2 and Bcl-xL for survival, and the co-delivery of siRNAs targeting these genes with doxorubicin using nucleolin-targeted mesoporous silica nanoparticles leads to an over 40-fold reduction in the IC_50_ value for doxorubicin [81]. Similarly, MDA-MB-468 TNBC spheroids exhibit resistance to gemcitabine and doxorubicin through mitochondrial stabilisation mediated by the RAS protein activator-like 2 (RASAL2) protein [83]. This protein causes the upregulation of BCL2 through activation of the Yes-associated protein (YAP) and cAMP response element-binding protein 1 (CREB1), which stabilises the mitochondrial membrane and prevents the release of cytochrome c [83]. The CREB1–BCL2 axis contributes to chemoresistance by suppressing intrinsic apoptotic signalling in TNBC spheroids that overexpress RASAL2 [83].

Beyond the suppression of intrinsic apoptotic pathways, chemoresistance in TNBC spheroids can be governed by the dampening of extrinsic apoptosis signalling. For example, the downregulation of the death receptor 4 (DR4) in BT-20 TNBC spheroids confers resistance to the tumour necrosis factor apoptosis-inducing ligand [84]. In addition, the resistance to TRAIL-induced apoptosis in MDA MB-231 spheroids is attributed to the sequestration of DR4 by the MCL-1 anti-apoptotic protein [82]. Relative to monolayers, the MCL-1 expression is higher in spheroids compared to that in monolayers, demonstrating that the propagation of cells into 3D aggregates upregulates anti-apoptotic proteins and contributes to chemoresistance in TNBC [82]. Beyond apoptotic resistance, CSC-driven pathways can contribute further to chemoresistance in TNBC spheroids.

### 2.7. Cancer Stem Cells and Pathway-Driven Chemoresistance

Various pathways can additionally contribute to chemoresistance in TNBC spheroids. These pathways are commonly regulated by enrichment in CSCs in spheroids derived from cell lines, resulting in the formation of specialised types of spheroids known as mammospheres [85]. These non-adherent mammospheres are commonly generated using serum-free medium, supplemented with growth factors such as EGF and fibroblast growth factor [85]. This allows for the enrichment of stem-cell-like cells that activate pathways that contribute to chemoresistance. A summary of the key pathways that contribute to chemoresistance in spheroids and mammospheres derived from TNBC cell lines is outlined in Table 1 below.

## 3. Strategies for Resensitising Chemoresistant TNBC Spheroids

The ability of multicellular spheroids to replicate the in vivo microenvironmental cues that contribute to chemoresistance makes these 3D constructs invaluable for assessing the effectiveness of novel strategies for overcoming resistance in TNBC. To address the multifactorial nature of chemoresistance in TNBC spheroids, various resensitisation strategies have been explored for overcoming the mechanisms of resistance identified in this review. To overcome resistance governed by the limitations of spatial diffusion, nanocarrier systems such as those including pH-responsive [94] and targeted nanoparticles [53] have been used to enhance the drug penetration within the heterogeneous and hypoxic TME in TNBC spheroids. Resistance to apoptosis has been circumvented through the co-delivery of chemotherapeutics with siRNAs targeting anti-apoptotic genes such as BCL-2 and BCL-xL [81]. Apigenin, a plant-derived flavonoid, has demonstrated dual activity by both promoting doxorubicin-induced apoptosis and downregulating efflux transporters, leading to the resensitisation of MDA-MB-231 spheroids [95]. Cancer-stem-cell-mediated resistance has been targeted using capivasertib (AZD5363), which resensitised stem cell spheres to doxorubicin and inhibited the doxorubicin-induced upregulation of the stemness markers CD133 and CD44 [96]. To overcome chemoresistance mediated by signalling pathways, dual inhibition strategies have been adopted. For example, a combination of pimasertib (AS-703026) and voxtalisib (SAR245409) has been used to target PI3K and p21-activated kinases 4/6 in MDA-MB-231 spheroids [97]. Crosstalk with the TME has been targeted in 4T1 and NIH/3T3 mouse embryonic fibroblast heterospheroids through the co-delivery of fucoxanthin and siTwist, which reduced the CAF activation and collagen content, thereby inhibiting spheroid growth [98]. Table 2 below summarises some of the therapeutic interventions used to resensitise chemoresistant TNBC spheroids, which are governed by various resistance mechanisms.

## 4. Limitations and Future Opportunities in Using Spheroids as Models for Studying Chemoresistance in TNBC Spheroids

Multicellular spheroids have shown great potential in mimicking in vivo conditions, allowing them to be widely used to probe the mechanisms contributing to chemoresistance in TNBC. However, the use of these models is not without limitations that limit the reliability of the data obtained. Direct comparisons of drug efficacy between monolayers were not widely reported in the reviewed studies, and only a limited number [56,69] reported the IC_50_ differences or fold changes in chemosensitivity. This was often because such comparisons were not the primary focus of the studies, or the comparisons were made relative to non-resistant spheroids rather than monolayers. Incorporating direct comparisons of the drug sensitivity in monolayers could aid in the elucidation of the role of 3D growth in chemoresistance. Additionally, most studies used the MDA-MB-231 cell line to generate spheroids. Although this is a useful model, the widespread adoption of spheroids from other TNBC cell lines [45] can improve the reliability of chemoresistance studies. Additionally, the traditional spheroid generation methods often result in the formation of spheroids with varying sizes and shapes, which can result in variability in drug responses [102]. MDA-MB-231 cells are mesenchymal-like and highly invasive, which may not reflect the behaviour of spheroids generated from TNBC cell lines of a different subtype [46]. For example, BT-20 cells exhibit a more epithelial phenotype and respond differently to drugs [103], highlighting the necessity of including a variety of TNBC cell lines when conducting chemoresistance studies. Additionally, the transition of cells from spheroids to monolayers introduces structural and metabolic changes that may take a long time to stabilise [104]. In commonly used static cultures, reduced oxygen and nutrient diffusion are accompanied by the accumulation of toxic waste, which hampers long-term culture [104]. In these static conditions, spheroids are often exposed to drugs after just 4 days of culture [17], a stage that might be too early for them to have developed the capacity to replicate in vivo functionality [104]. In various studies, spheroids are exposed between Day 3 and Day 5 of spheroid culture [105,106], despite evidence that key physiological features such as hypoxia can sometimes emerge only after longer culturing periods [107]. Additionally, some cells may require up to 18 days after trypsinisation to re-establish in vivo-like physiological and ultrastructural traits [24]. This highlights the need for culturing platforms that allow for long-term culturing of spheroids, enabling the examination of drug responses in mature spheroids.

To this end, dynamic cell culturing platforms such as rotating wall vessel clinostat bioreactors have been used to circumvent the limitations associated with the traditional spheroid generation methods. Clinostats have some advantages over other spheroid generation techniques. Firstly, constant rotation ensures thorough mixing of nutrients and O_2_ throughout the cell culture media without damaging the cells due to low shear forces [37]. These low shear forces result in the reproducible generation of multicellular spheroids [108]. Secondly, clinostat-based bioreactor systems such as the CelVivo ClinoStar™ allow for long-term spheroid cultures for up to 35 days, enabling the characterisation of cellular and molecular changes over time [109]. This system facilitates real-time monitoring of the effects of drugs in spheroids over extended periods, enabling reproducible testing of chronic treatments and resistance studies using physiologically stable spheroids. Using this approach, cisplatin-resistant MDA-MB-231 spheroids have recently been grown in the clinostat system, allowing for an experimental period of up to 22 days post-seeding [110].

The TME is heterogeneous, comprising neoplastic cells and surrounding stromal cells such as CAFs and TAMs [111]. However, many spheroid models lack vascularisation, which limits the nutrient diffusion, drug penetration, and overall physiological relevance [112]. These limitations are more pronounced in static systems, while dynamic culturing platforms allow for improved perfusion and transport [54]. Additionally, many studies using spheroids for the investigation of chemoresistance in TNBC still rely on homotypic spheroids composed of only cancerous cells. Consequently, the role of heterotypic interactions in mediating chemoresistance may not be fully recapitulated. To recapitulate this in vivo heterogeneity, these stromal cells are incorporated into spheroids, allowing for the investigation of tumour crosstalk as a mechanism of chemoresistance in TNBC spheroids [73]. However, the widespread adoption of heterospheroids in experimental settings can be limited by the inherent challenges associated with co-culturing diverse cell types. In heterospheroids, different cell types can sort unpredictably. For example, increasing the fibroblast ratio causes fibroblasts to cluster centrally in spheroids, hampering the recapitulation of the widespread distribution of cell types observed in the in vivo TME [113].

Bioprinting has emerged as a promising strategy for circumventing the limitations associated with the standard co-culture models and investigating mechanisms of chemoresistance [114]. For example, cisplatin-resistant spheroids created using extrusion-based bioprinting overexpress drug resistance markers such as the GRP78 chaperone and ABCG2 [115]. Bioprinting enables precise control over the composition and spatial distribution of the cells and the ECM within 3D constructs. This is particularly useful in experimental settings where tumour crosstalk is being probed as a mechanism of chemoresistance [114]. However, the use of extrusion-based techniques is limited by high shear stress, that can compromise cell viability and alter the cellular phenotype [116]. Additionally, the relatively low spatial resolution of these techniques restricts accurate reconstruction of the intricate tumour architecture [117]. To circumvent the limitation of scalability in the generation of bioprinted spheroids, the use of multi-nozzle systems enables simultaneous placement, achieving speeds 10× faster than those in the conventional methods [118].

Since spatial heterogeneity and limited drug diffusion contribute to the development of TNBC spheroids, it is necessary to adopt analytical tools that provide spatially resolved information. Phase contrast microscopy is a tool that is generally readily available in most experimental settings, as it is a relatively simple and cheaper technique compared to other imaging modalities [17]. This technique has been used for the characterisation of spheroid morphology and volume [17], and when coupled with automation algorithms, phase contrast microscopy becomes an invaluable tool in high-throughput evaluations of growth kinetics and drug toxicity [38]. However, the spatial zonation of spheroids cannot be inferred using phase contrast microscopy; thus, histological and immunohistology techniques have to be conducted to investigate this phenomenon. The main drawbacks of this approach are that (i) the sample preparation introduces experimental artefacts and (ii) histological dyes have a low spatial resolution [119]. This necessitates the use of more advanced techniques to probe spatial variations in cell viability, proliferation, and protein expression.

The analytical workflows for probing protein and gene expression have been optimised for 2D cell cultures and often involve sample lysis, resulting in the loss of information regarding spatially resolved data, which is a major limitation when analysing spheroids [120]. Although confocal microscopy has been widely used to image fluorescently labelled spheroids, it is constrained by a limited imaging depth of approximately 100 μm, restricting the analysis to the outer cell layers. This poses a major limitation in large spheroids (>150–300 μm), where light scattering and absorption prevent accurate visualisation of the inner spheroid regions [121]. Additionally, fluorescence-based assays in spheroids can be limited by uneven dye penetration, photobleaching, and intrinsic cellular autofluorescence. This can compromise signal stability, often resulting in quantification errors and inaccurate interpretation of spatially resolved spheroid imaging data [122]. Various techniques have been developed to circumvent these limitations and are summarised in Table 3.

Despite increasing advances in spheroid growth and analytical techniques, a persistent gap remains between in vitro findings and clinical outcomes. Spheroids lack the biological complexity of in vivo tumours, including vascular networks, immune system components, variable cell types contributing to more complex signalling pathways, and the systemic environment, which collectively influence the pharmacokinetics and drug response in patients [112]. Moreover, variations in the methodologies, coupled with challenges in reproducibility [40], introduce inconsistencies that limit the predictive value of spheroids for translational relevance. Bridging this gap will require continuous refinement through extensive characterisation of the spheroid models and the use of standardised protocols to improve reproducibility. Finally, enhancing the multicellular and microenvironmental complexity of spheroids by incorporating dynamic or biomimetic platforms will improve their utility in elucidating the mechanisms of chemoresistance in TNBC and strengthen their translational relevance in preclinical research.

## 5. Conclusions

Relative to traditional monolayer culturing systems, multicellular spheroids offer several advantages for drug testing, rendering them a reliable platform for probing the mechanisms of chemoresistance in TNBC. A plethora of studies have utilised TNBC spheroids to identify these mechanisms, which include spatial heterogeneity, hypoxia, extracellular matrix remodelling, tumour–stroma crosstalk, drug efflux, apoptotic resistance, and cancer stem cell signalling. These multifactorial and interlinked mechanisms reflect the in vivo TME that spheroids are able to recapitulate, rendering them valuable models for studying chemoresistance. However, limitations such as low reproducibility, a lack of heterogeneity, and limited analytical techniques can preclude the widespread adoption of spheroids when investigating the mechanisms of chemoresistance in TNBC. Further development of spheroid models, including the incorporation of stromal components and advanced analytical techniques, will enhance their utility for preclinical drug discovery.

## Figures and Tables

**Table 1:** Signalling pathways contributing to chemoresistance in TNBC spheroids and mammospheres.

Pathway	Pathway Function	Mechanism of Resistance	Model Used	Reference
ERK1/2 and ERK5	Regulates the EMT and survival	ERK5 activation regulates the survival of anoikis-resistant spheroids, contributing to chemoresistance.	Spheroids (MDA-MB-231, BT-549)	[86]
Hippo (YAP/TAZ)	Promotes tissue-specific progenitor cells during renewal and regeneration and facilitates cell proliferation	YAP/TAZ maintains stemness properties, regulates redox homeostasis, and modulates mitochondrial dynamics, leading to chemoresistance to paclitaxel.	Mammospheres (MDA-MB-231, MDA-MB-468, and 4T1)	[87]
Notch1	Maintains a CSC phenotype	Notch signalling promotes resistance to targeted or cytotoxic therapies by enriching a small population of CSCs.	Mammospheres (MDA-MB-231, BT20)	[88]
STAT3	Maintains stemness	STAT3 enhances CSC survival and promotes chemoresistance to doxorubicin.	Mammospheres (BT-549)	[89]
USP22	Promotes glycolysis, the EMT, and CSC traits	USP22 promotes glycolysis via c-Myc deubiquitination, which enhances stemness and the EMT phenotype, leading to chemoresistance.	Mammospheres (BT-549, MDA-MB-231)	[90]
WAVE3/β-catenin	Stabilises β-catenin; sustains CSC survival	WAVE3 prevents β-catenin degradation and maintains stemness after exposure to cisplatin, doxorubicin, and paclitaxel.	Mammospheres (MDA-MB-231)	[91]
Wnt/β-catenin	Is involved in CSC maintenance and cell proliferation	Hyperactive Wnt signalling and the downregulation of tumour suppressor genes cause high levels of self-renewal and dysregulated proliferation, leading to chemoresistance.	Spheroids (MDA-MB-231)	[92]
Wnt/FZD8	Is involved in CSC maintenance and growth	Wnt signalling through the FZD8 and LRP6 receptors leads to the enrichment of cisplatin-resistant CSCs.	Mammospheres (MDA-MB-468, MDA-MB-231, CRL-2335)	[93]

Abbreviations: CSC—cancer stem cell; EMT—epithelial–mesenchymal transition; ERK 1/2/5—extracellular signal-regulated kinases 1, 2, and 5; FZD8—Frizzled class receptor 8; LRP6—low-density lipoprotein receptor-related protein 6; STAT3—signal transducer and activator of transcription 3; TAZ—transcriptional co-activator with PDZ-binding motif; USP22—ubiquitin-specific peptidase 22; WAVE3—Wiskott–Aldrich syndrome protein family member 3; YAP—Yes-associated protein; MDA-MB-231, BT-549, MDA-MB-468, BT20, and CRL-2335—human TNBC cell lines; 4T1—murine TNBC cell line.

**Table 2:** Resensitisation strategies to overcome chemoresistance in TNBC spheroids.

Chemoresistance Mechanism	Intervention Drug(s)	Resensitisation Mechanism	Reference(s)
Limited diffusion	Doxorubicin and miR-34a-loaded hybrid micelles	Improved drug penetration and distribution throughout the spheroids.	[53]
Hypoxia	TH-302 (hypoxia-activated prodrug)	TH-302 is activated in hypoxic regions, releasing a DNA crosslinker that targets doxorubicin-resistant hypoxic cells.	[49]
Spatial heterogeneity	Paclitaxel, everolimus, trametinib	This combination targets cells with multiple phenotypes.	[52]
ECM remodelling	Fucoxanthin and twist siRNA (siTwist)	Reduces the deposition of collagen, leading to better drug penetration.	[98]
Tumour crosstalk (TAMs)	Cetuximab-conjugated gold nanorods + NIR irradiation	Causes polarisation of pro-tumoural TAMs (M2-like) to an antitumoural phenotype (M1-like)	[75]
Tumour crosstalk (CAFs)	Fucoxanthin and siTwist	Targeting the Twist gene (important for CAF activation) and using FX (multi-target effects) resensitise the tumour microenvironment.	[98]
Drug efflux	Doxorubicin and ATRA	ATRA inhibits efflux pumps, leading to an increased intracellular doxorubicin concentration.	[99]
	Bacopaside II	Bacopaside II increases intracellular doxorubicin accumulation by inhibiting ABC transporters like ABCC3, which are overexpressed in resistant TNBC spheroids.	[100]
Resistance to apoptosis	Apigenin and doxorubicin	Apigenin sensitises TNBC spheroids to doxorubicin-induced apoptosis by triggering DNA damage, activating the caspase-9-mediated intrinsic apoptotic pathway, and increasing caspase-3 activity.	[95]
CSCs and signalling	ASA + metformin + oseltamivir phosphate	The reduction in the CD44/CD24 ratio and ALDH1A1 expression reverses stemness.	[101]
	PD0325901 (MEK/ERK inhibitor) PI-103 (PI3K/AKT inhibitor)	Inhibition of MAPK and PI3K activation reverses paclitaxel resistance in spheroids.	[74]

Abbreviations: ABC, ATP-binding cassette; ABCC3, ATP-binding cassette subfamily C member 3; ATRA, all-trans retinoic acid; CAF, cancer-associated fibroblast; CSC, cancer stem cell; ECM, extracellular matrix; FX, fucoxanthin; MAPK, mitogen-activated protein kinase; MEK, MAPK/ERK kinase; M1, classically activated (pro-inflammatory) macrophage phenotype; M2, alternatively activated (anti-inflammatory or pro-tumoural) macrophage phenotype; NIR, near-infrared; PI3K, phosphoinositide 3-kinase; siRNA, small interfering RNA; siTwist, siRNA targeting Twist; TAM, tumour-associated macrophage; TNBC, triple-negative breast cancer.

**Table 3:** Techniques for achieving a good spatial resolution for multicellular spheroids.

Technique	Applications	Principle	Reference(s)
Consecutive Cryosectioning	Immunofluorescence imaging of spheroids	Spheroids are embedded into a freezing medium, frozen, and sectioned into thin slices using a cryotome; this enables high-resolution imaging of the internal architecture with improved section integrity and a reduced layer overlap.	[123]
Expansion Microscopy	Nanoscale-resolution imaging of tumour spheroids	Spheroids are embedded into a swellable polymer gel, enzymatically digested the sample, and physically expanded to achieve super-resolution imaging with conventional microscopy.	[124]
Light-Sheet Fluorescence Microscopy	Three-dimensional imaging of large spheroids	Spheroids are illuminated with a thin sheet of light for optical sectioning. Each plane is captured with a camera to rapidly acquire high-resolution volumetric fluorescence images with minimal photobleaching.	[125]
MALDI MSI	Spatial metabolomic/lipidomic profiling	Matrix-assisted laser desorption/ionisation is performed on thin spheroid sections; enhanced MALDI with trapped ion mobility is used to obtain high-resolution maps of lipids and metabolites across the spheroid.	[126,127]
MRI	Non-invasive, label-free 3D characterisation of spheroid clusters	Used 3T MRI with quantitative mapping is used to assess spheroids’ structure, viability, and extracellular matrix composition over time without disrupting the sample.	[128]
Multiphoton Microscopy	Multimodal imaging and therapeutic monitoring	The two-photon luminescence and X-ray contrast properties of plasmonic nanocapsules with gold nanoislands and fluorescent payloads are used to image spheroids.	[129]
Optical Clearing	Three-dimensional imaging of large spheroids	Reduces light scattering by matching the refractive indices, allowing deeper and clearer imaging of spheroids.	[130]
Optical Coherence Tomography	Label-free 3D live imaging of spheroids	Uses low-coherence interferometry to generate cross-sectional images of the spheroids.	[131]
Serial Trypsinisation	Spatial proteomics, transcriptomics, and metabolomics	Enzymatically disassociates various layers in spheroids, which can be isolated and analysed using downstream assays.	[132]
Diffusion Smart-seq3	Spatial single-cell transcriptomics in spheroids	Diffuses dye into the spheroid, labelling cells by their radial position; sorted cells undergo deep Smart-seq3xpress single-cell RNA-seq to map the gene expression from the core to the periphery.	[51]

Abbreviations: 3D, three-dimensional; 3T; 3 Tesla; MALDI, matrix-assisted laser desorption/ionisation; MALDI MSI, matrix-assisted laser desorption/ionisation mass spectrometry imaging; MALDI-2, post-ionisation MALDI; MRI, magnetic resonance imaging; RNA, ribonucleic acid; T1/T2, MRI relaxation times (T1: longitudinal, T2: transverse).

## Data Availability

No new data were generated in this study.

## Abbreviations

The following abbreviations are used in this manuscript:

2D	Two-dimensional
3D	Three-dimensional
ABC	ATP-binding cassette
ASA	Acetylsalicylic acid
ALDH1A1	Aldehyde dehydrogenase 1 family member A1
ATP	Adenosine triphosphate
ATRA	All-trans retinoic acid
Bcl-2	B-cell lymphoma 2
Bcl-xL	B-cell lymphoma-extra-large
BCRP	Breast cancer resistance protein
CD	Cluster of differentiation
CAF	Cancer-associated fibroblast
CREB1	cAMP response element-binding protein 1
CSC	Cancer stem cell
CXCL12	C-X-C motif chemokine ligand 12
CXCR4	Chemokine receptor Type 4
DNA	Deoxyribonucleic acid
DR4	Death receptor 4
ECM	Extracellular matrix
EMT	Epithelial–mesenchymal transition
ERK1/2	Extracellular signal-regulated kinases 1 and 2
ERK5	Extracellular signal-regulated kinase 5
FZD8	Frizzled class receptor 8
FX	Fucoxanthin
HIF	Hypoxia-inducible factor
IL	Interleukin
LRP6	Low-density lipoprotein receptor-related protein 6
MAPK	Mitogen-activated protein kinase
MEK	MAPK/ERK kinase
MALDI	Matrix-assisted laser desorption/ionisation
MALDI MSI	MALDI mass spectrometry imaging
MCL-1	Myeloid cell leukaemia 1
MRI	Magnetic resonance imaging
NIR	Near-infrared
O_2_	Oxygen
pCR	Pathological complete response
PD0325901	MEK/ERK inhibitor
P-gp	P-glycoprotein
PI-103	PI3K/AKT inhibitor
PI3K	Phosphoinositide 3-kinase
RASAL2	RAS protein activator-like 2
RNA	Ribonucleic acid
siRNA	Small interfering RNA
siTwist	SiRNA targeting Twist
STAT3	Signal transducer and activator of transcription 3
TAM	Tumour-associated macrophage
TAZ	Transcriptional co-activator with PDZ-binding motif
TGF-β	Transforming growth factor-beta
TME	Tumour microenvironment
TNBC	Triple-negative breast cancer
USP22	Ubiquitin-specific peptidase 22
WAVE3	Wiskott–Aldrich syndrome protein family member 3
YAP	Yes-associated protein
T1/T2	MRI relaxation times
Smart-seq3	Single-cell RNA sequencing technique
